# Availability of Diagnostic Services and their Impact on Patient Flow in Two Brazilian Referral Centres of Breast Cancer Treatment

**DOI:** 10.31557/APJCP.2020.21.2.317

**Published:** 2020

**Authors:** Tácila Thamires de Melo Santos, Lorena Sofia dos Santos Andrade, Milena Edite Case de Oliveira, Kedma Anne Lima Gomes, Tiago Almeida de Oliveira, Mathias Weller

**Affiliations:** 1 *Postgraduate Program in Public Health, *; 2 *Department of Statistics, State University of Paraíba (UEPB), Campina Grande-Paraíba, Brazil. *

**Keywords:** Breast cancer, treatment delay, private and public health care provider

## Abstract

**Background:**

System delay (SD) is a leading cause of advanced stage of disease and poor prognosis among Brazilian breast cancer patients.

**Methods:**

Cox regression and Kaplan-Meier analysis were used to identify variables that contributed to SD among 128 breast cancer patients. Time intervals between first medical consultation and treatment initiation were compared among patients of two referral centres: Patients of a referral centre with outsourced (FAP), respectively, integrated (HNL) diagnostic services.

**Results:**

Women who used a specialized private clinic at the beginning of patient flow had an 2.32 fold increased chance (95% CI: 1.17 - 4.60; p = 0.016) of hospital admission within 90 days after first medical consultation, compared to women who used a public health care provider (HCP). Of 73 and 34 patients of the FAP hospital and the HNL, respectively, 10 (13.7%) and 11 (32.5%) used one HCP prior to hospital admission (p = 0.000). The median time between first medical consultation and treatment initiation was 150 days. The median time between first medical consultation and hospital admission was 136.0 and 52.0 days for patients of the FAP hospital, respectively the HNL (p < 0.050). The median time between first medical consultation and diagnostic mammography was 36.5 and 23.0 days for patients from the FAP hospital and the HNL (p < 0.050).

**Conclusions:**

Usage of public diagnostic services was associated with increased SD, whereas the usage of private diagnostic services diminished it. The usage of a lower number of HCPs accelerated patient flow.

## Introduction

Breast cancer is the leading cause of death from cancer among women worldwide, and the burden of disease is progressively shifting from developed to developing countries (Torre et al., 2016). Despite a slight decline of breast cancer mortality rates, Latin American countries remain plagued by high mortality-to-incidence ratios (Carioli et al., 2018). The total number of deaths due to breast cancer in Latin American countries is expected to double between 2008 and 2030 to 73,542 cases, and it has been reported that 30% to 40% of patients present at advanced stages (III and IV; Justo et al., 2013). In Brazil, the largest Latin American country, the national cancer institute (INCA) predicted 59,700 new cases in 2018, with breast cancer contributing 29.5% to all cancer cases among women (INCA, 2018). In southern regions of Brazil, including urban centres such as São Paulo and Rio de Janeiro, breast cancer incidence has remained stable or has declined in recent years (INCA, 2005; INCA, 2018). This is in sharp contrast to the north-eastern region of Brazil, where demographic changes, characterised by increased life expectancy of women, led to an increase of breast cancer incidence: between 2005 and 2018, the incidence grew from 27.23 to 38.84 new cases per 100,000 women (INCA, 2005; INCA, 2018).

One of the major problems contributing to increased mortality rates for breast cancer in developing countries is delay of treatment (Unger-Saldaña, 2014). Time delays that are determined by behavioural characteristics of patients have been defined as patient delays, whereas system-dependent time delays of diagnosis and treatment have been defined as SD (Ramirez et al., 1999; Richards et al., 1999; Caplan, 2014; Unger-Saldaña, 2014). A time delay of more than 90 days between initial diagnosis by first medical consultation because of symptoms, discovery of a tumour by mammography, or clinical breast examination (CBE) and initiation of treatment, was defined as SD (Ramirez et al., 1999; Richards et al., 1999; Williams, 2015). Treatment of breast cancer is complex and requires several diagnostic and therapeutic procedures. Delays anywhere on the diagnostic and treatment pathway can significantly increase the mortality rate. In this context, SD was associated with advanced stage (Stage III and IV) of disease and poor prognosis (Caplan, 2014; Unger-Saldaña, 2014; Williams, 2015). A meta-analysis of 87 studies indicated that an SD of > 3 - 6 months after initial diagnosis was associated with a 12% decrease of 5-year survival compared to patients who received treatment within a 3-month period (Richards et al., 1999). In 34 studies of SD, poor disease management, access barriers, diagnostic delays and poor communication were the most often-identified causal factors of SD (Freitas and Weller, 2015). Additionally, socio-economic variables, including income, education and ethnic origin were associated with differences in time-to-treatment and, due to their influence on speed of treatment decisions by physicians, specific histopathological characteristics of disease may also lead to SD (Freitas and Weller, 2015).

Most Brazilian studies of SD were performed in the southern, south-eastern and central-western regions of the country (Trufelli et al., 2007; Souza et al., 2008; Trufelli et al., 2008; Rezende et al., 2009; Lourenço, 2010; Soares et al., 2012; Barros et al., 2013; Oshiro et al., 2014; Guerra et al., 2015; Souza et al., 2015; Trufelli et al., 2015; Lopes et al., 2017). By contrast, only a few studies of SD focused on populations in the Brazilian northeastern region that is experiencing increasing disease incidence (Cavalcanti et al., 2012; Paiva et al., 2015; Ferreira et al., 2017). Breast cancer patients who use the Brazilian public health care system (Sistema Único de Saúde, or SUS), must receive treatment for disease within 60 days of receipt of an anatomic-pathological diagnosis (Ministério da Saúde, 2017). On the one hand, this law is neither applicable to patients who receive their anatomic-pathological diagnosis after surgery, nor does it guarantee initiation of treatment within three months after initial diagnosis of a breast tumour. On the other hand, the law is applicable to patients whose anatomic-pathological diagnosis is based on a biopsy prior to treatment initiation. 

There is an ongoing debate in Brazil regarding the privatization of the public health care system (McGregor et al., 2017). The Brazilian SUS is a public health care system that provides access to health facilities for all Brazilians. On the one hand, access is free and public health care providers guarantee treatment of any disease. On the other hand, mismanagement of funds, corruption, underinvestment, poor quality and SD remain unresolved problems for the SUS (McGregor et al., 2017; Mendes et al., 2018). This is one of the main reasons for a growing private health care sector that exists in parallel with the public system: About 25% of the Brazilian population have access to private health services, financed by individual health insurance (Malta et al., 2017; McGregor et al., 2017). Furthermore, in the case of disease, patients have the option to combine self-financed private and free public health services for diagnosis and treatment. In addition, the SUS is a decentralized system that allows cooperation between the public and private health sectors, affording the possibility of contracts between municipal governments and private health care providers (McGregor et al., 2017). In the case of cancer diagnosis, this means that there are public referral centres for treatment that offer all diagnostic services within a hospital, whereas there are others that outsource these diagnostic services in part or in full. 

In the present study, patient flow was compared between two public breast cancer referral centres in the northeast region of Brazil. We asked if the number of used diagnostic services affected patient flow. Furthermore, in one referral centre, diagnostic services were performed within the hospital, whereas in the other one these services were outsourced to private laboratories. Our study aimed to elucidate possible differences in patient flow between these systems, including integrated and outsourced diagnostic services. Underlying socio-demographic variables that affect SD from the first medical consultation until hospital admission and treatment initiation were analysed.

## Materials and Methods

*Study population*

The data sampling protocol was reviewed and approved by the Brazilian National Research Ethics Committee (CONEP; Nr.: CEP-UEPB: 63083816000005187). Written informed consent was obtained from each participant to participate in this study and to publish data.

Patient data were obtained from two Brazilian cancer treatment referral centres: the Hospital Napoleão Laureano (HNL) in João Pessoa and the hospital Fundação Assistencial da Paraíba (FAP) in Campina Grande. We refer to each as HNL and FAP, respectively. João Pessoa, the capital of the state of Paraíba, has about 800,000 inhabitants and is located on the coast (IGBE, 2010). Campina Grande, with about 400,000 inhabitants, is the second most-populated urban centre in Paraíba and is located about 120 km away from the capital in the interior of the state (IGBE, 2010). Paraíba has mixed-ethnic population of indigenous, African and European ancestry. 

*Diagnosis of breast cancer in the referral centres HNL and FAP*

The HNL has an integrated pathology section. Therefore, anatomic-histopathological and immunohistochemical exams can be performed within the referral centre. Furthermore, diagnostic mammography also can be performed in the referral centre. The FAP, by contrast, has neither a pathology section nor did their patients undergo diagnostic mammography at the referral centre. In the case of the FAP, diagnostic mammography and anatomic-histopathological and immunohistochemical exams were financed privately or by the SUS, but were performed by private laboratories and medical centres in Campina Grande. Notably, both referral centres in this study, medical records data were not yet digitized and often incomplete.

*Data sampling*

Data were sampled between January and October 2016. Sampling included 89 and 39 patients from FAP and HNL, respectively. Only patients who received treatment within this time span in one of the two- referral centres, FAP or HNL, were included in the study. Patients with recurrence of disease and patients with cognitive problems were excluded. Patients with in situ tumours were also excluded.

Clinical and histopathological data were obtained from medical records. All data regarding patient flow, including the time interval between first medical consultation and treatment initiation and diagnostic procedures, were obtained from medical records. In addition, information regarding patient flow was also obtained from personal interviews of all 128 women (see below). If personal information and medical records data regarding specific time points or sequence of used health services were discordant, we relied on medical record data. If the time for usage of a particular health service was neither available by interview, nor was documented in the medical record, the corresponding patient was excluded from analysis of time intervals that included this health service as a starting or endpoint of a defined time interval. 

A structured questionnaire was administered to patients within the chemotherapy and radiotherapy units of both hospitals. All interviews were performed by one of the authors. The questionnaire included information regarding socio-economic data, including age, ethnic origin, education level, civil status, the income and health insurance status. Educational level was defined as follows: Without any schooling was defined as “analphabetic”. Incomplete and complete elementary education meant less than nine years or nine years of basic school education, respectively. Incomplete and complete high school education of women meant less than 12 and 12 years of school education, respectively. High school or college meant that the women had more than 12 years schooling or university education. Minimum wage and multiple values were used to characterize income. This is a popular and well-known method used to define economic level among low- and middle-class subjects. The minimum wage or less was defined as “low” income, whereas multiples of the minimum wage were defined as “high” income. The minimum wage in 2018 was R$954.00/month (US$281.60/month; 20 April 2018). Information regarding ethnic origin was obtained by self-report of participating women. Women were asked about quality of health care accessibility and types, as well as frequency of health service use before and after diagnosis of their disease. Additionally, women were asked about adherence to the mammography-screening program. Of all 128 women, 56 (43.7%) stated they underwent regular mammography, and in two (1.6%) patients, disease was detected by screening mammography.

*Statistical analysis*

Data were tabulated in Excel® software (version 10; MICROSOFT, 2010) and all statistical analyses were performed with R software (version 3.4.3; R Core Team, 2017) using the Therneau T package (version 2.38; R Core Team, 2015). Cox regression analysis was performed to determine hazard ratios (HR) and confidence intervals (CI) of variables. Values of p were determined by likelihood-ratio tests. As described by Collet (1994), the final model of Cox regression analysis was adjusted for all variables with a value of p ≤ 0.1 in univariate analysis. Median values were calculated to compare total time intervals between patients of the FAP hospital and the HNL, using the Kruskal-Wallis and the Mann-Whitney U test. In the Kaplan-Meier analysis, time intervals of >90 days and >60 days were defined as events. Kaplan-Meier analysis was applied to calculate the risk of an event within a determined time interval. Estimated mean values and 75th percentiles were used to compare time delays in days among patients of the FAP and HNL.

## Results

*Variables that affect system delay*

Breast cancer patients were on average 54.3 (StD = 24.4) years old and age ranged from 30 to 93 years. General characteristics of patients and results of univariate Cox regression analysis with respect to time interval between the first medical consultation and hospital admission are summarized in Table 1. Of the 128 patients, 45 (35.2%) and 83 (64.8%) were ≤ 49 years and ≥ 50 old, respectively (Table 1). Caucasian and mixed ancestry, were reported by 58 (45.7%) and 53 (41.7%), respectively (Table 1). Analphabetic and incomplete elementary education, were reported by 6 (4.9%) and 47 (38.2%), respectively, whereas 18 (14.6%) reported high school diploma or college degree (Table 1). Of all women, 61 (48.8%) were married and 70 (57.4%) had low income (Table 1). When asked about health insurance status, 104 (87.9%) women reported having no private health insurance (Table 1). Of 121 women, 23 (19%) claimed to have had poor access to health care services before their diagnosis (Table 1). Of 82 patients with known tumour hormone receptor status, 61 (74.4%) were oestrogen-positive and 19 (23.2%) were HER2-positive (Table 1). Histological exams of 91 patients revealed that 57 (62.6%) had advanced (III and IV) stages of disease (Table 1).

Patients of the HNL had a greater chance (HR = 2.67; 95% CI: 0.46-1.05; p = 0.001) of hospital admission within 90 days after first medical consultation than did patients of the FAP (Table 1). Furthermore, high income and having private health insurance increased the chances (HR = 2.19; 95% CI: 1.24-3.84; p = 0.007 and HR = 2.54; 95% CI: 0.39-1.32; p = 0.005, respectively) of hospital admission within 90 days, compared to low income and having no private health insurance (Table 1). Women who exclusively used public or both, public and private HCP before and after detection of disease until hospital admission, had a 2.4-fold (HR = 0.42; 95% CI: 0.22-0.82; p = 0.010 and 4.4-fold (HR = 0.23; 95% CI: 0.08-0.60; p = 0.002) lower chance of timely hospital admission, respectively, compared to women who used only private HCP (Table 1). If asked about frequency of utilization of primary public HCP before diagnosis of disease, those women who used it each month had a 2.9-fold (HR = 0.34; 95% CI: 0.16- 0.75; p = 0.007) lower chance of timely hospital admission compared to women who never used it (Table 1). Women who used a specialized private clinic at the beginning of patient flow had a 2.4-fold increased chance (95%CI: 1.25-4.51; p = 0.008) of hospital admission within 90 days of the first medical consultation compared to women who used a public primary HCP (Table 1). The socio-economic variables age, ethnic origin, educational level, civil status and adherence to mammography screening program were not significantly associated with hospital admission within 90 days of the first medical consultation (Table 1). Furthermore, the data did not indicate any significant association between stage of disease, hormone or HER2 receptor status and speed of patient flow (Table 1). 

To identify independent variables, an adjusted Cox regression model was created (Table 2). In this adjusted model, patients of the HNL had an increased chance (HR= 2.08; 95% CI: 1.09 - 3.94; p = 0.026) of hospital admission within 90 days of the first medical consultation compared to patients of the FAP (Table 2). Women who never used primary public HCP had an increased chance of hospital admission within 90 days, compared to those who used it each month (HR= 0.29; 95% CI: 0.12 - 0.71; p = 0.006) or a minimum of once per year (HR= 0.37; 95% CI: 0.15 - 0.91; p = 0.029). Furthermore, women who initially used a specialized private clinic at the beginning of patient flow had a 2.3-fold increased chance (95%CI: 1.17 - 4.60; p = 0.016) of timely hospital admission compared to women who used a public primary HCP (Table 2).

*Comparison of variables between patients from the FAP and the HNL*

Socio-demographic variables were not significantly different among patients of the FAP and HNL (not shown). Comparison of private versus public HCP use by patients before hospital admission, did not reveal differences between FAP and HNL: Eleven (13.3%) and six (15.8%) out of 83 and 38 patients of the FAP and HNL, respectively, used private health care providers exclusively before and after detection of disease (p = 0.828). Furthermore, 32 (35.9%) out of 89 and 14 (36.9%) out of 38 patients of the FAP and HNL, respectively, used a specialized private clinic at beginning of patient flow (p = 0.780). 

*Time intervals between first medical consultation and hospital admission*

Of 83 patients treated in the FAP, 69 (83.1%) underwent an anatomic-histopathological exam before hospital admission, whereas in the HNL, 15 (68.2%) out of 22 underwent an exam after hospital admission (p = 0.000). 

To elucidate differences of patient flow between FAP, and HNL, distinct time intervals were analysed (Table 3). The median time between first medical consultation and hospital admission of 118 patients was 120.5 days (Table 3). The median time between first medical consultation and treatment initiation of 122 patients was 150 days (Table 3). Significant differences of time intervals among patients indicated faster patient flow in the HNL, compared to the FAP hospital: The median time between first medical consultation and realization of diagnostic mammography was 36.5 and 23.0 days for patients from the FAP hospital and the HNL (p < 0.050; Table 3). The median time between first medical consultation and hospital admission to the FAP hospital and the HNL was 136.0, respectively 52.0 days (p < 0.050; Table 3). Patients of the FAP hospital and the HNL who delayed >90 days, had a median time of 180.0 and 163.0 days between first medical consultation and treatment initiation (p= 0.041; Table 3). The median time between result of anatomic-pathological exam and treatment initiation was 56.0 days and in 35 (44.3%) out of 79 cases this time interval exceeded 60 days (Table 3). 

*Number of HCP used by patients of the FAP and the HNL*

Patients of the FAP and HNL used on average 2.47 (StD = 0.97) and 2.03 (StD = 1.06) public and/or private HCP, respectively, before hospital admission (p = 0.038; Table 4). Of 73 and 34 patients of the FAP and HNL, respectively, 10 (13.7%) and 11 (32.5%) had used one HCP before hospital admission (p = 0.000; Table 4). Mean distance between home and referral centre was 61.95 km (StD = 75.25) and 182.21 km (StD = 196.08) for patients from the HNL and FAP hospital, respectively (p = 0.000).

**Table 1:** Socio –Economic Characteristics of Patients (N= 128) and Cox Regression Analysis of Variables. Hazard Ratios (HR) Were Calculated for the Chance of Hospital Admission within 90 Days after First Medical Consultation

Variable	N (%)	HR (95%CI)	p
Referral centre			
FAP	89 (69.5%)	Ref.	
HNL	39 (30.5%)	2.67 (0.46-1.05)	0.001
Age (years)			
< 50 years	45 (35.2%)	Ref.	
≥ 50 years	83 (64.8%)	0.00 (0- )	0.997
Ethnic origin			
Caucasian	58 (45.7%)	Ref.	
African	14 (11.0%)	0.50 (0.16-1.69)	0.267
Indigenous	2 (1.6%)	1.21 (0.68-2.14)	0.519
Mixed ancestry	53 (41.7%)	0.03 (3.34-278.35)	0.997
Missing	1		
Educational level			
Analphabetic	6 (4.9%)	Ref.	
Incomplete elementary education	47 (38.2%)	0.46 (0.13-1.59)	0.223
Complete elementary education	19 (15.5%)	0.35 (0.08-1.47)	0.152
Incomplete high school education	9 (7.3%)	0.85 (0.20-3.54)	0.821
Complete high school education	24 (19.5%)	0.72 (0.19-2.56)	0.609
High school or college	18 (14.6%)	0.70 (0.19-2.59)	0.592
Missing	5		
Civil status			
No union	29 (23.2%)	Ref.	
Married	61 (48.8%)	0.83 (0.41-0.68)	0.676
Stable union	7 (5.6%)	0.00 (0.00- )	0.997
Widow	17 (13.6%)	1.33 (0.62-2.84)	0.462
Divorced	11 (8.8%)	0.69 (0.25-1.99)	0.496
Missing	3		
Income			
Low	70 (57.4%)	Ref.	
High	52 (42.6%)	2.19 (1.24-3.84)	0.007
Missing	6		
Private health insurance			
No	104 (87.9%)	Ref.	
Yes	19 (16.1%)	2.54 (0.39-1.32)	0.005
Missing	5		
Easy access to health care			
No	23 (19.0%)	Ref.	
Yes	98 (81.0%)	1.28 (0.60-2.73)	0.518
Missing	7		
HCP used before and after detection of disease until hospital admission			
Private	17 (14.0%)	Ref.	
Public	78 (64.5%)	0.42 (0.22-0.82)	0.010
Public and private	26 (21.5%)	0.23 (0.08-0.60)	0.002
Missing	7		
Variable	N (%)	HR (95%CI)	p
Frequency of utilization of primary public HCP before diagnosis of disease			
Never	26 (20.2%)	Ref.	
At minimum one time each year	37 (28.7%)	0.50 (0.23-1.07)	0.074
Every six months	19 (14.7%)	0.59 (0.25-1.40)	0.236
Every three months	4 (3.1%)	0.28 (0.04- 2.18)	0.225
Every month	42 (32.6%)	0.34 (0.16- 0.75)	0.007
First HCP used by women at beginning of patient flow			
Public primary HCP	53 (41.1%)	Ref.	
Specialized private clinic	46 (35.7%)	2.37 (1.25-4.51)	0.008
Community hospital	26 (20.2%)	1.82 (0.82-4.04)	0.144
Public health care centre	2 (1.6%)	2.26 (0.30-17.17)	0.428
Regular participation on mammography screening program			
No	50 (41.7%)	Ref.	
Yes	70 (58.3%)	1.16 (0.65-2.05)	0.617
Missing	8		
Oestrogen receptor status			
Negative	21 (25.6%)	Ref.	
Positive	61 (74.4%)	0.99 (0.50-1.98)	0.977
Missing	22		
Progesterone receptor (PR) status			
Negative	33 (40.2%)	Ref.	
Positive	49 (59.8%)	1.57 (0.49-1.55)	0.570
Missing	22		
HER-2 receptor (HER2) status			
Negative	63 (76.8%)	Ref.	
Positive	19 (23.2%)	1.09 (0.56-2.31)	0.728
Missing	22		
TNM stage			
I	5 (5.5%)	Ref.	
II	29 (31.9%)	0.70 (0.15-3.22)	0.644
III	47 (51.6%)	1.33 (0.31-5.63)	0.697
IV	10 (11.0%)	1.57 (0.34-7.28)	0.563
Missing	37		

**Table 2:** Hazard Ratios (HR) and Confidence Intervals (CI) in an Adjusted Cox Regression Model. The model of adjusted variables refers to hospital admission within 90 days after first medical consultation

Variable	N (%)	HR (CI 95%)	P
Referral centre			
FAP	74 (83.1%)	Ref.	
HNL	15 (16.9%)	2.08 (1.09 - 3.94)	0.026
Frequency of utilization of primary public health care providers before diagnosis of disease			
Never	26 (20.3%)	Ref.	
At minimum one time each year	37 (28.9%)	0.37 (0.15 - 0.91)	0.029
Every six months	19 (14.9%)	0.55 (0.22 - 1.37)	0.197
Every three months	4 (3.1%)	0.14 (0.01 - 1.29)	0.083
Every month	42 (32.8%)	0.29 (0.12 - 0.71)	0.006
First service used by women at beginning of patient flow			
Primary public HCP	53 (41.7%)	Ref.	
Specialized private clinic	46 (36.2%)	2.32 (1.17 - 4.60)	0.016
Community hospital	26 (20.5%)	0.63 (0.21 - 1.91)	0.419
Public health care centre	2 (1.6%)	0.76 (0.08 - 7.06)	0.812

**Table 3:** Time Intervals are Shown in Days for Descriptive and Kaplan-Meier Analysis. Numbers, percentages and mean and median values of Kaplan-Meier analysis refer to patients who delayed > 90 days and > 60 days for a determined time interval. The 75th percentile of Kaplan-Meier analysis defined the time (in days) after which 75% of these patients delayed. Diagnostic results were defined as the date of the result of the clinic-histopathological exam

	All					Delay >90 days				
	N	Mean	75%	Median	P	NDELAY	Mean	75%	Median (95%CI)	P
First medical consultation - realization of diagnostic mammography										
All	65	65.3	73.5	35		18	163.3	197	129	
		(s= 84.1)				(27.70%)	(s= 25.9)		(87.7- 170.3)	
FAP	48	69.1	66.8	36.5	<0.050	13	171.5	155	133	0.78
		(s= 90.7)				(27.10%)	(s= 35.3)		(81.5- 184.5)	
HNL	17	54.7	105.5	23		5	141.8		129	
		(s= 63.1)				(29.40%)	(s= 19.5)		(87.7- 170.3)	
First medical consultation - hospital admission										
All	118	163.04	213.0.0	120.5		74	230.15	276.5	192	
		(s= 138.0)				(62.70%)	(s= 110.6)		(165.7- 218.4)	
FAP	87	324.8	229	136	<0.050	62	235.9	284	192	0.38
		(s= 1082.9)				(71.30%)	(s= 18.1)		(167.9- 216.1)	
HNL	31	79.8	107.8	52		12	200.7	267	174	
		(s= 80.9)				(38.70%)	(s= 20.8)		(82.3- 265.7)	
First medical consultation - treatment initiation										
All	122	178.8	222.8	150		90	222.1	275	176	
		(s= 178.8)				(73.80%)	(s= 13.5)		(159.3-192.7)	
FAP	86	193.2	231	156	≥0.050	65	236.6	284	180	0.041
		(s= 143.6)				(74.70%)	(s= 17.3)		(168.7- 191.3)	
HNL	35	147.4	193	138		25	184.5	207	163	
		(s= 94.1)				(71.40%)	(s= 17.0)		(146.8- 179.2)	
All						Delay >60 days				
Diagnostic mammography - result of anatomic-pathological exam										
All	80	156.6	182.3	86		50	233.5	274	147	
		(s= 227.2)				(62.50%)	(s= 258.8)		(110.0- 184.0)	
FAP	61	168.9	176.5	83	≥0.050	39	247.5	288	154	0.537
		(s= 253.2)				(63.90%)	(s= 46.3)		(114.8- 193.2)	
HNL	19	116.9	234	100		11	183.9	245	146	
		(s= 103.1)				(57.90%)	(s= 25.7)		(27.3- 264.7)	
	All					Delay >90 days				
	N	Mean	75%	Median	P	NDELAY	Mean	75%	Median (95%CI)	P
Biopsy – result of anatomic-pathological exam										
All	103	10.6	14	7			-			-
		(s= 9.6)								
FAP	82	11	14	7	≥0.050		-			-
		(s= 10.2)								
HNL	20	8.1	13.8	8						
		(s= 5.4)								
Result of anatomic-pathological exam - treatment initiation										
All	79	64.7	86	56		35	100.5			
		(s= 167.8)				(44.30%)	(s= 7.8)			
FAP	66	64.6	85.3	56	≥0.050	37	101.4			0.853
		(s= 47.9)				(43.90%)	(s= 9.3)			
HNL	13	65.1	95.5	57		6	96.3			
		(s= 34.1)				(46.20%)	(s= 7.5)			

ALL, All patients who delayed and not delayed; FAP, Patients from the FAP; HNL, Patients from the HNL

**Table 4:** Number of HCP (NHCP) Used by Patients (N= 107) before Hospital Admission

	FAP (N= 73)	HNL (N= 34)
Mean*	2.47 (StD= 0.97)	2.03 (StD= 1.06)
NHCP**	N (%)	
1	10 (13.7%)	11 (32.4%)
2	31 (42.5%)	15 (44.1%)
3	23 (31.5%)	6 (17.6%)
4	6 (8.2%)	1 (2.9%)
5	3 (4.1%)	-
6	-	1 (2.9%)

* p, 0.038; **p, 0.000; StD, Standard deviation

## Discussion

Present data showed that women who had never used primary public HCP before diagnosis had an increased chance of hospital admission within 90 days after the first medical consultation compared to those ones who used it each month and at a minimum once per year. Furthermore, women who used a specialised private clinic instead of a primary public HCP at beginning of patient flow had a more than two-fold greater chance of hospital admission within 90 days. In agreement with present results, several previous Brazilian studies reported SD in the public health care system (Rezende et al., 2009; Souza et al., 2012; Barros et al., 2013; Oshiro et al., 2014). Brazilian studies performed in the states of Minas Gerais and in Rio de Janeiro suggested that usage of public versus private was associated with advanced stage of disease and decreased chance of five-year survival (Rezende et al., 2009; Guerra et al., 2015). In a study that included data from 56,094 women in various Brazilian regions, authors reported increased SD with respect to the time between diagnosis and treatment initiation for public referrals compared to private ones (Medeiros et al., 2015). In a recent study performed in the state of Ceará, northeast Brazil, the median time between diagnosis and hospital admission was 39 to 71.5 days for patients referred by the private and public health system, respectively (Ferreira et al., 2017). Similar, in the present study, the median time between the diagnostic result of the anatomic-pathological exam and treatment initiation at public referral centres was 56.0 days, but in 35 (44.3%) out of 79 cases, it exceeded the obligatory 60 days guaranteed by the public health care system.

According to previous Brazilian studies, long waiting times for specialized medical consultations and diagnostic exams were among the main problems in cancer treatment by public health care providers (Gebrim, 2016; Ferreira et al., 2017). These long waiting times can often be the result of a lack of specialized services: Cavalcanti et al., (2012) for example, reported waiting times of up to 30 days for specialized services after detection of first symptoms. Other earlier studies performed in the states of Rio de Janeiro and Minas Gerais reported median and mean waiting times of 6.5 and >6 months, respectively, between the first medical consultation and diagnostic confirmation (Rezende et al., 2009; Soares et al., 2012). Furthermore, there were no guidelines for referrals or requests for subsidiary exams by health professional (Gebrim, 2016; Ferreira et al., 2017). Authors of a previous study that focused on the time interval between screening mammography and treatment initiation of patients in São Paulo identified missing coordination of patient flow and social assistance of patients as organizational problems and principal reasons for SD (Trufelli et al., 2008).

To the best of our knowledge this was the first Brazilian study that aimed to compare patient flow among different public referral centres of breast cancer treatment. The time interval between the first medical consultation and hospital admission was significantly shorter for patients of the HNL than for patients of the FAP. The number of HCP used by patients before admission to the HNL was significantly lower, compared to those used by patients before admission to the FAP. In agreement with present results, a recent Mexican study associated diagnostic delay with increased number of HCP, used before admission to a specialized health care centre (Unger-Saldaña et al., 2018). According to the INCA and the Ministry of Health, women who experience breast cancer symptoms should visit a primary HCP for clinical breast examination (Migowski et al., 2018). From this primary service, patients should be referred to diagnostic mammography and an anatomic-pathological exam. Private HCP can perform diagnostic services and they are financed by the SUS. After diagnostic confirmation of breast cancer, patients should then be referred to units or centres with high complexity of cancer treatment (Unidades/Centros de Alta Complexidade Oncológica; UNACON/CACON; Migowski et al., 2018). Diagnostic mammography and anatomic-histopathological exams are often not offered by the same HCP. Therefore, the official concept requires in many cases consultation of at minimum four health care providers until treatment initiation. This contrasts with the present results that indicated that concentration of health services could accelerate patient flow. 

In the case of the HNL diagnostic services, were concentrated in the referral centre, whereas in the FAP, these services were outsourced. Most patients of the HNL underwent anatomic-histopathological and immunohistochemical exams in the referral centre. This could mean that outsourcing of the diagnostic service in the FAP contributed to SD. In the outsourced system, longer administrative procedures and waiting times for results from private laboratories at medical centres could contribute to SD. The number of patients, who had used private HCP before and after hospital admission at beginning of patient flow, was very similar in the HNL and the FAP. However, differences of SD between HNL and FAP could also be explainable by distinct patient flow within private HCPs used by both groups of patients.

The most severe limitation of the present study was the small amount of data. This prevented resolution of detailed differences of time-to-treatment between both referral centres. This low resolution was further aggravated, by missing data. Due to small amount of data obtained it was not possible to compare SD among patients of both referral centres who used exclusively public diagnostic services. Similar, patient flow of private HCP was not compared between both groups of patients. The study did not determine why SD was less pronounced for patients who used private HCP than for those ones who used only public HCP. Possible reasons remain speculative and were not analysed in more detail.

In conclusion, we found greater SD for patients who used public HCP before diagnosis of breast cancer and at beginning to patient flow than for women who never used public HCP before diagnosis and who started patient flow in a specialized private clinic. Present data indicated that a lower number of HCP used before hospital admission diminished SD, whereas a high number of used HCP increased SD. Future studies should focus on the comparison between integrated and outsourced diagnostic services and their effect on patient flow. Furthermore, SD also should be compared among private services. 

## References

[B1] Barros FA, Uemura G, Macedo JLS (2013). Tempo Pará Acesso ao Tratamento do Câncer de mama não Distrito Federal, Brasil Central. Rev Bras Ginecol Obstet.

[B2] Caplan L (2014). Delay in breast cancer: Implications for stage at diagnosis and survival. Front Public Health.

[B3] Carioli G, Malvezzi M, Rodriguez T (2018). Trends and predictions to 2020 in breast cancer mortality: Americas and Australasia. Breast J.

[B4] Cavalcanti LPG, Simões PSF, Silva MRR, Galdino PNR (2012). Assistência em mastologia em uma unidade de referência do Sistema Único de Saúde no Ceará, Brasil. Rev Bras Canc.

[B5] Collett D (1994). Modelling survival data medical research.

[B6] Ferreira NAS, Carvalho SMF, Valenti VE (2017). Treatment delays among women with breast cancer in a low socio-economic status region in Brazil. BMC Womens Health.

[B7] Freitas AGQ, Weller M (2015). Patient delays and system delays in breast cancer treatment in developed and developing countries. Ciência Saúde Col.

[B8] Gebrim, LHA (2016). Detecção precoce do câncer de mama no Brasil. Cad Saúde Pública.

[B9] Guerra MR, Silva GA, Nogueira MC (2015). Sobrevida por câncer de mama e iniquidade em saúde. Cad Saúde Pública.

[B10] Justo N, Wilking N, Jönsson B, Luciani S, Cazap E (2013). A review of breast cancer care and outcomes in Latin America. Oncologist.

[B11] Lopes TCR, Gravena AAF, Demitto MO (2017). Delay in diagnosis and treatment of breast cancer among women attending a reference service in Brazil. Asian Pac J Canc Prev.

[B12] Lourenço AV (2010). Women cancer prevention and pharmaceutical contribution. Braz J Pharm Sci.

[B13] Malta DC, Stopa SR, Pereira CA (2017). Private health care coverage in the Brazilian population, according to the 2013 Brazilian National Health Survey. Ciência Saúde Col.

[B14] Medeiros GC, Bergmann A, Aguiar SS, Thuler LCS (2015). Análise dos determinantes que influenciam o tempo para o início do tratamento de mulheres com câncer de mama no Brasil. Cad Saúde Pública.

[B17] McGregor AJ, Siqueira CE, Zaslavsky AM, Blendon RJ (2017). Do elections matter for private-sector healthcare management in Brazil? An analysis of municipal health policy. BMC Health Serv Res.

[B22] Oshiro ML, Bergmann A, Silva RG (2014). Câncer de mama avançado como evento sentinela para avaliação do programa de detecção precoce do câncer de mama no centro-oeste do Brasil. Rev Bras Canc.

[B23] Paiva CJK, Cesse EAP (2015). Aspectos relacionados ao atraso no diagnóstico e tratamento do câncer de mama em uma unidade hospitalar de pernambuco. Rev Bras Canc.

[B24] Ramirez AJ, Westcombe AM, Burgess CC (1999). Factors predicting delayed presentation of symptomatic breast cancer: a systematic review. Lancet.

[B25] Rezende MCR, Koch HA, Figueiredo JA, Thuler LCS (2009). Causas do retardo na confirmação diagnóstica de lesões mamárias em mulheres atendidas em um centro de referência do Sistema Único de Saúde no Rio de Janeiro. Rev Bras Ginecol Obstet.

[B26] Richards MA, Westcombe AM, Love SB, Littlejohns P, Ramirez AJ (1999). Influence of delay on survival in patients with breast cancer: a systematic review. Lancet.

[B27] Soares PBM, Filho SQ, Souza WP (2012). Characteristics of women with breast cancer seen at reference services in the North of Minas Gerais. Rev Bras Epidemiol.

[B28] Souza VO, Grando JPS, Couto Filho JO (2008). Tempo decorrido entre o diagnóstico de câncer de mama e o início do tratamento, em pacientes atendidas no Instituto de Câncer de Londrina (ICL). Rev Bras Med.

[B29] Souza CB, Fustinoni SM, Amorim MHC (2015). Estudo do tempo entre o diagnóstico e início do tratamento do câncer de mama em idosas de um hospital de referência em São Paulo, Brasil. Ciência Saúde Col.

[B30] Torre LA, Siegel RL, Ward EM, Jemal A (2016). Global cancer incidence and mortality rates and trends--An update. Cancer Epidemiol Biomarkers Prev.

[B31] Trufelli DC, Bensi CG, Pane CEV (2007). Onde está o atraso? Avaliação do tempo necessário para o diagnóstico e tratamento do câncer de mama nos serviços de oncologia da Faculdade de Medicina do ABC. Rev Bras Mastol.

[B32] Trufelli DC, Miranda VC, Santos MBB (2008). Análise do atraso no diagnóstico e tratamento do câncer de mama em um hospital público. Rev Assoc Med Bras.

[B33] Trufelli DC, Matos LL, Santi PX, Del Giglio A (2015). Adjuvant treatment delay in breast cancer patients. Rev Assoc Med Bras.

[B34] Unger-Saldaña K (2014). Challenges to the early diagnosis and treatment of breast cancer in developing countries. World J Clin Oncol.

[B35] Unger-Saldaña K, Ventosa-Santaulària D, Miranda A, Verduzco-Bustos G (2018). Barriers and explanatory mechanisms of delays in the patient and diagnosis intervals of care for breast cancer in Mexico. Oncologist.

[B36] Williams F (2015). Assessment of breast cancer treatment delay impact on prognosis and survival: a look at the evidence from systematic analysis of the literature A Pilot Study. J Cancer Biol Res.

